# Correlations Between Tumor Mutation Burden and Immunocyte Infiltration and Their Prognostic Value in Colon Cancer

**DOI:** 10.3389/fgene.2021.623424

**Published:** 2021-02-16

**Authors:** Zhangjian Zhou, Xin Xie, Xuan Wang, Xin Zhang, Wenxin Li, Tuanhe Sun, Yifan Cai, Jianhua Wu, Chengxue Dang, Hao Zhang

**Affiliations:** ^1^Department of Oncology, The Second Affiliated Hospital of Xi’an Jiaotong University, Xi’an, China; ^2^Department of Surgical Oncology, The First Affiliated Hospital of Xi’an Jiaotong University, Xi’an, China

**Keywords:** colon cancer, tumor mutation burden (TMB), immunocytes infiltration, prognosis prediction, bioinformatics

## Abstract

**Background:**

Colon cancer has a huge incidence and mortality worldwide every year. Immunotherapy could be a new therapeutic option for patients with advanced colon cancer. Tumor mutation burden (TMB) and immune infiltration are considered critical in immunotherapy but their characteristics in colon cancer are still controversial.

**Methods:**

The somatic mutation, transcriptome, and clinical data of patients with colon cancer were obtained from the TCGA database. Patients were divided into low or high TMB groups using the median TMB value. Somatic mutation landscape, differentially expressed genes, and immune-related hub genes, Gene Ontology and KEGG, gene set enrichment, and immune infiltration analyses were investigated between the two TMB groups. Univariate and multivariate Cox analyses were utilized to construct a prognostic gene signature. The differences in immune infiltration, and the expression of HLA-related genes and checkpoint genes were investigated between the two immunity groups based on single sample gene set enrichment analysis. Finally, a nomogram of the prognostic prediction model integrating TMB, immune infiltration, and clinical parameters was established. Calibration plots and receiver operating characteristic curves (ROC) were drawn, and the C-index was calculated to assess the predictive ability.

**Results:**

Missense mutations and single nucleotide polymorphisms were the major variant characteristics in colon cancer. The TMB level showed significant differences in N stage, M stage, pathological stage, and immune infiltration. CD8^+^ T cells, activated memory CD4^+^ T cells, activated NK cells, and M1 macrophages infiltrated more in the high-TMB group. The antigen processing and presentation signaling pathway was enriched in the high-TMB group. Two immune related genes (CHGB and SCT) were identified to be correlated with colon cancer survival (HR = 1.39, *P* = 0.01; HR = 1.26, *P* = 0.02, respectively). Notably, the expression of SCT was identified as a risk factor in the immune risk model, in which high risk patients showed poorer survival (*P* = 0.04). High immunity status exhibited significant correlations with immune response pathways, HLA-related genes, and immune checkpoint genes. Finally, including nine factors, our nomogram prediction model showed better calibration (C-index = 0.764) and had an AUC of 0.737.

**Conclusion:**

In this study, we investigated the patterns and prognostic roles of TMB and immune infiltration in colon cancer, which provided new insights into the tumor microenvironment and immunotherapies and the development of a novel nomogram prognostic prediction model for patients with colon cancer.

## Introduction

Colon cancer is the most common neoplasm in the digestive system, contributing to approximately 1.1 million new cases and 550,000 deaths in 2018, which makes it the third ranking cancer based on incidence and the second leading cancer based on mortality among all malignances (Bray et al., 2018). The epidemiological characteristics show a modernity manner with a higher incidence in developed countries, such as European countries and Australia/New Zealand, than in developing countries (Brody, 2015; Bray et al., 2018). However, in China, the incidence of colon cancer exhibits a mixture of profiles with huge differences between urban and rural areas (Chen et al., 2016). Due to different lifestyles and socioeconomic statuses, the incidence of colon cancer in urban areas is higher than that in rural areas, while the mortality remains similar (Pan et al., 2017). With improvements in early screening methods and therapeutic strategies, new cases and death rates have decreased in elderly people over the past 10 years. Notably, for people < 50 years old, the incidence has increased for unknown reasons, which indicates that more investigations and research in young adults are warranted (Siegel et al., 2017).

For advanced patients who lose curable surgery opportunities, systematic or multidisciplinary therapeutic strategies, including chemotherapy, targeted therapy, and immunotherapy could be considered to improve the prognosis (Dienstmann et al., 2015; Auclin et al., 2017; Wu, 2018). To avoid immunosurveillance, tumor cells always upregulate the expression of immune checkpoint-related genes, such as programmed cell death protein-1 (PD-1) and cytotoxic T lymphocyte antigen 4 (CTLA4), during tumor development, which will cause T cell anergy or apoptosis (Leach et al., 1996; Iwai et al., 2002; Chan et al., 2019). However, with the discovery of improved survival in metastatic melanoma by ipilimumab, a CTLA-4 antibody, immunotherapy provides a new strategy for advanced metastatic tumors (Hodi et al., 2010). Recently, overall survival was increased in different tumors by administering immune checkpoint blockade therapy (ICB), including urothelial cancer, renal cell carcinoma, non-small cell lung cancer (NSCLC), and hematologic malignancies (Ansell et al., 2015; Borghaei et al., 2015; Motzer et al., 2015; Rosenberg et al., 2016; Goodman A. et al., 2017). To predict the response to immunotherapy, tumor mutation burden (TMB) is used as an evaluating marker (Samstein et al., 2019). Tumors with high TMB levels lead to more mutation derived neoantigens, inducing higher immunogenicity across diverse tumors (Goodman A. M. et al., 2017).

In colorectal cancer (CRC), mutation profiles can be divided into two types, mismatch repair deficient or high levels of microsatellite instability (dMMR-MSI-H) and mismatch repair proficient or low levels of microsatellite instability (pMMR-MSI-L) (Ganesh et al., 2019). The dMMR-MSI-H CRCs exhibit high levels of TMB and activated CD8^+^ cytotoxic T cell infiltration, which results in survival improvement with ICB treatments (Llosa et al., 2015; Ganesh et al., 2019). However, patients with pMMR-MSI-L CRC show no response to current ICBs. To date, the potential mechanisms of TMB and immunocyte infiltration in colon cancer development are still controversial. In this study, we analyzed somatic mutations and immunocytes in filtration patterns of colon cancer based on data from The Cancer Genome Atlas (TCGA) database and constructed a novel nomogram model to estimate the prognosis of colon cancer patients, which might be helpful to explore proper therapeutic strategies for patients with colon cancer.

## Materials and Methods

### Databases

We downloaded all available data on somatic mutations, transcriptome profiles, microsatellite instability (MSI) status, and clinical information of colon cancer separately from the TCGA database^1^. In total, 399 samples with somatic mutation data were analyzed to show the mutation landscapes of colon cancer. The transcriptome profiles of 398 colon cancer samples and 39 normal samples were extracted to explore immune infiltration characteristics and related genes or pathways. In addition, the clinical information of 452 patients with colon cancer was obtained, including age, race, sex, therapies, pathological stage, AJCC-TNM stages, and survival status. Then, 343 matched samples from mutation data, transcriptome data, and clinical data with the same sample ID were enrolled to analyze the TMB level, differentially expressed genes or pathways, immune infiltration status, and potential clinical application in prognostic prediction or therapeutic management. The workflow of this study is shown in Supplementary Figure 1.

### Tumor Mutation Burden Analysis

To explore the mutation landscapes of colon cancer, the somatic mutation data were processed and analyzed by R software (version 4.0.2) with the “maftools” package^2^. TMB was defined as the total number of somatic mutations including somatic mutations, insertion-deletion mutations, coding, and base replacement of per million bases. The colon cancer patients were separated into the low-TMB and high-TMB groups using the median value of TMB. To analyze the correlations between TMB and clinicopathological factors of patients with colon cancer, we merged the TMB data with corresponding clinical information. The Wilcoxon rank-sum test was utilized for comparisons between two groups of clinical variables.

### Microsatellite Instability (MSI) Analysis

The MSI status (MSI-H, MSI-L, and MSS) of colon cancer samples was obtained by R software with the “TCGAbiolinks” package. The genomes of cancer samples were tested by five mononucleotide markers (BAT25, BAT26, NR21, NR24, and MONO27) (Bacher et al., 2004). Samples were identified as MSI-H when >40% of the mononucleotide markers were altered, MSI-L when <40% of the mononucleotide markers were altered, and MSS when no mononucleotide marker was altered.

### Gene Expression and Functional Enrichment Analysis

Before analyzing the gene expression differences between the low- and high-TMB groups of colon cancer patients, we combined the TMB data with the corresponding transcriptome profiles. Background correction, normalization, and visualization of raw transcriptome data were performed by R software with the “limma” package. Differentially expressed genes (DEGs) were determined between the low- and high-TMB groups by cutoff values of | log_2_(Fold Change)| > 1 and *P*-value < 0.05. The expression of the top 20 DEGs in various samples is shown in the heat map constructed by R software with the “pheatmap” package.

To explore the functions and signaling pathways of genes that were differentially expressed between the two TMB groups, we performed a Gene Ontology (GO) function analysis and Kyoto Encyclopedia of Genes and Genomes (KEGG) pathway enrichment analysis using R software with the “org.Hs.eg.db,” “clusterProfiler,” “enrichplot,” and “ggplot2” packages.

### Gene Set Enrichment Analysis (GSEA)

To further investigate the enrichment of gene functions and signaling pathways between the low- and high-TMB groups, GSEA was performed based on the JAVA8 platform. We selected the “c2.cp.kegg.v7.0.symbols.gmt” gene sets as reference sets, which were obtained from the MSigDB database^3^. The significant enrichment of GO functions and KEGG pathways was considered only with a normalized *P*-value < 0.05 and a FDR *q* value < 0.25.

### Immune Infiltration Analysis Between Low- and High-TMB Groups

To analyze the immune infiltration of each colon cancer sample, the relative fractions of immunocytes were calculated using the CIBERSORT algorithm (Newman et al., 2015). Quantification of each immunocyte subtype among colon cancer samples was based on the gene expression signatures of 22 different subtypes of immunocytes, LM22, which included gene sets from B cells (memory and naive B cells, and plasma cells), T cells (naive CD4^+^ T cells, activated and resting memory CD4^+^ T cells, CD8^+^ T cells, regulatory T cells, follicle-assisted T cells and γδT cells), NK cells (resting and activated NK cells) and myeloid cells (resting and activated dendritic cells, monocytes, M0-2 macrophages, resting and activated mast cells, neutrophils, and eosinophils). The transcriptome data of colon cancer samples were submitted to the CIBERSORT web portal^4^, with the algorithm using the default signature matrix at 1,000 permutations. The distributions of immunocytes in the low- and high-TMB groups were determined by R software with the “pheatmap” package. The Wilcoxon rank-sum test was exploited to compare the differential fractions of immune infiltration between these two groups, which were exhibited with *P*-values by R software with the “vioplot” package.

### Immunity Profile Analysis of Colon Cancer by Single Sample Gene Set Enrichment Analysis (ssGSEA)

In this study, we also investigated the immunity profiles of every colon cancer sample based on transcriptome data, which included the type of immunocytes and immune related pathways, fractions of infiltrated immunocytes, expression of human leukocyte antigen (HLA) genes, and immune checkpoint genes. Gene expression landscapes of immunocytes and immune related pathways from both innate and specific immunity were analyzed and enriched by ssGSEA with the “GSVA” R package (Barbie et al., 2009). Based on immune enrichment scores calculated by the “Consensus Cluster Plus” package in ssGSEA, colon cancer samples were divided into the low- and high- immunity groups. Then, the tumor purity, ESTIMATE scores, immune scores, and stromal scores were analyzed and compared by the “estimate” package and the Mann–Whitney *U* test between low and high immunity groups.

### Immune Related Hub Gene Analysis and Validation by the TIMER Database

To further investigate immune related hub genes in colon cancer, the expression level of immune related hub genes among 32 different tumors and correlations with immunocyte infiltration were analyzed and validated by the TIMER database^5^. The “Diff Exp” module was used to estimate the hub gene expression between tumors and matched normal tissues of different types of cancer. In addition, the “Gene” module was applied to calculate the correlation between hub gene expression and immunocyte infiltration levels, including B cells, CD4^+^ T cells, CD8^+^ T cells, neutrophils, macrophages, and dendritic cells.

### Construction of a Prognostic Risk Model by Immune-Related Genes

To identify immune related genes that were differentially expressed between different TMB groups, we obtained 1,811 immune-related genes from the Immunology Database and Analysis Portal (Immport). After overlapping with the DEGs from our TCGA cohort by R software with the “VennDiagram” package, univariate and multivariate Cox regression analyses were conducted to obtain the coefficient (β) of immune related hub genes. The risk score was calculated as follows: risk score = β_1_ × expression of gene 1 + β_2_ × expression of gene 2 + …… + β_n_ × expression of gene n. Then, patients with colon cancer were divided into low- and high-risk groups by the median risk score as the cutoff value. Kaplan–Meier analysis was conducted to compare the survival difference between the low- and high-risk groups.

### Construction and Evaluation of the Nomogram Model for Patients With Colon Cancer

In our study, the survival probability of colon cancer patients from the TCGA database was estimated by the nomogram model integrating the TMB, immune infiltration and immune-related gene signatures with clinicopathologic features, which was also performed with the “rms” package in R software. For nomogram establishment, each level of factors (like male and female) is assigned a score, which reflects their influence degree on the outcome variable (death) in the nomogram model. Their influence on the survival of patients was quantified as the size of the regression coefficient in the multivariate Cox regression analysis. Then each score of each factor is added to get the total score. Finally, through the function transformation between the total score and the probability of the outcome event, the predicted survival probability of each patient is calculated. Calibration plots, ROC curves, and the C-index were generated to assess the performance of the nomogram model. The survival probability prediction and actual survival rate are displayed on the *y*-axis and *x*-axis separately in the calibration graphs, in which the 45-degree dotted line indicates an ideal prediction. The bootstrapping method was used as an internal validation to decrease the bias of the C-index’s predictive ability.

### Statistical Analysis

All statistical analyses as well as the visualizations were performed by R software (version 4.0.2) with related R packages. Correlations between the TMB, immune infiltrations, and MSI status were estimated using the Chi-square test and Fisher’s exact test. Other detailed statistical methods are mentioned in the above sections. A *P*-value < 0.05 was considered statistically significant.

## Results

### Tumor Mutation Profiles and MSI Status in Colon Cancer

**FIGURE 1:**
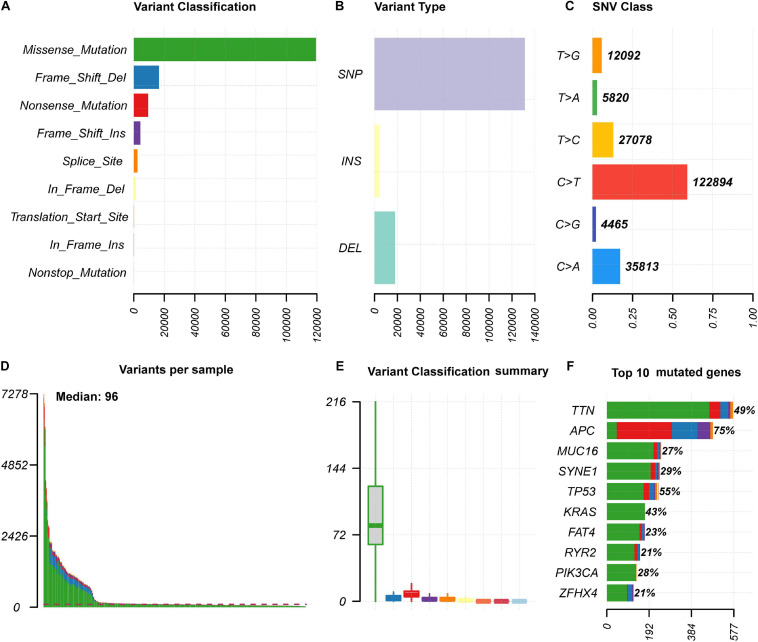
The summary information of somatic mutation in colon cancer samples from the TCGA database. **(A)** Frequency of different mutation classification. **(B)** Frequency of mutational variant types. **(C)** Frequency of SNV class. **(D,E)** TMB level of each colon cancer samples. **(F)** Top 10 mutated genes in colon cancer samples. SNP, single nucleotide polymorphism; INS, insertion; DEL, deletion; SNV, single nucleotide variants.

We analyzed somatic mutation data of 399 colon cancer samples from the TCGA database. The “maftolls” R package was used to visualize the mutation annotation format of colon cancer. In general, missense mutations were the most frequent type among nine different types of mutations (Figure 1A). For different mutation variant types, single nucleotide polymorphisms (SNPs) showed a higher frequency than deletion (DEL) and insertion (INS) mutations, and C > T was the major type of single nucleotide variant classification (SNV, Figures 1B,C). In each sample, the number and classification of variants were analyzed and are shown in a boxplot (Figures 1D,E). Furthermore, we analyzed specific mutated genes among colon cancer samples and found the top 10 mutated genes, including TTN (49%), APC (75%), MUC16 (27%), SYNE1 (29%), TP53 (55%), KRAS (43%), FAT4 (23%), RYR2 (21%), PIK3CA (28%), and ZFHX4 (21%), which might play important roles in colon cancer biological processes (Figure 1F).

**FIGURE 2:**
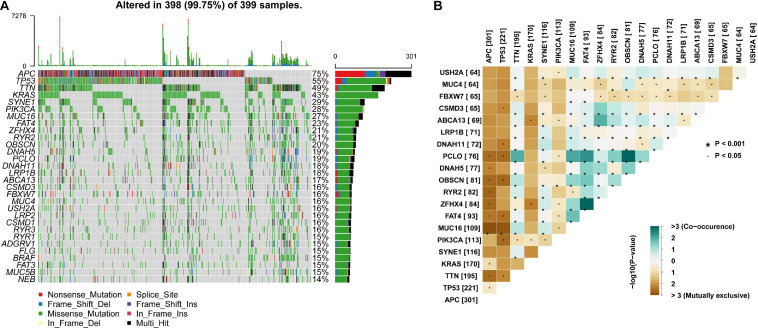
Landscape and potential associations between mutation genes. **(A)** Waterfall plot showing mutation profiles of each gene in each colon cancer sample. The left panel shows the genes ordered by mutation frequency, which is listed in the right panel. **(B)** The co-occurrence or exclusive associations between top 20 mutation genes.

In addition, mutation details of each colon cancer sample are shown in a waterfall plot (Figure 2A), in which we could analyze different mutation types for each individual gene involved in colon cancer progression. Among these highly altered genes, the associations between each pair of genes are exhibited in Figure 2B, which shows that co-occurrence existed between PCLO and OBSCN, as well as ZFHX4 and FAT4. However, the associations between MUC16 and APC or TP53 were mutually exclusive (Figure 2B).

Furthermore, we obtained the MSI data of 459 patients with colon cancer from the TCGA database. Based on the category method of MSI, a total of 373 samples were classified as MSI-H and 86 samples were classified as MSI-L/MSS. Samples with matched TMB and MSI profiles were analyzed to estimate the correlation between TMB and MSI of colon cancer. As shown in Table 1, those patients with high TMB were more likely to be MSI-H (*P* < 0.001).

**TABLE 1:** Correlations between tumor mutation burden, immune infiltrations, and microsatellite instability status in colon cancer.

	MSI-H (%)	MSI-L/MSS (%)	χ^2^	*P*-value
TMB-H (%)	72 (18.2)	126 (31.8)		
TMB-L (%)	11 (2.8)	187 (47.2)	56.72	<0.001
Immunity-H (%)	59 (16.6)	197 (55.4)		
Immunity-L (%)	3 (0.8)	97 (27.2)	20.09	<0.001

*MSI-H, microsatellite instability-high; MSI-L/MSS, microsatellite instability-low/stable; TMB-H, tumor mutation burden-high; TMB-L, tumor mutation burden-low; Immunity-H, immunity-high; Immunity-L, immunity-low.*

### The Correlation Between TMB and Colon Cancer Clinicopathological Parameters

**FIGURE 3:**
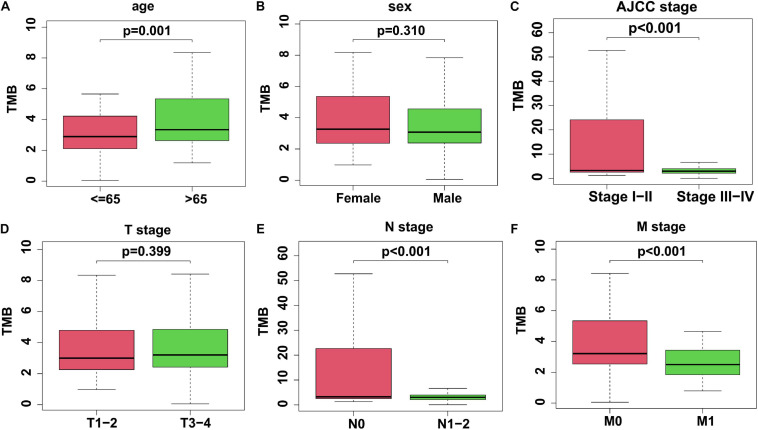
The association between TMB and clinicopathological factors. **(A)** Elderly colon cancer patients > 65 years old exhibited a higher TMB level. **(B)** There was no significant difference of TMB level in different genders. **(C)** Colon cancer patients with Stage III-IV exhibited a lower TMB level. **(D)** No significant difference of TMB levels in different T stages. **(E,F)** TMB level was lower in colon cancer patients with N1-2 stage or M1 stage. TMB, tumor mutation burden.

Due to the consideration of the TMB as a marker for tumor mutational status, we analyzed the TMB levels combined with different clinicopathological factors. After matching with mutation data, clinical data, and transcriptome data, a cohort consisting of 343 colon cancer samples was used in this study to investigate the differences between various TMB levels (Table 2). Colon cancer patients with higher clinical stages (Stage III-IV), advanced N stage (N1-2), and M1 stage showed significantly lower TMB levels. However, elderly patients aged > 65 years exhibited higher TMB levels. Unfortunately, we could not find any correlation between sex or T stage and TMB (Figure 3).

**TABLE 2:** Clinicopathological characteristics of colon cancer patients from TCGA database.

Characteristics		Patients Number (%)
Age	≦65	141 (41.11)
	¿65	202 (58.89)
Sex	Male	177 (51.60)
	Female	166 (48.40)
T stage	*T*_1_	9 (2.62)
	*T*_2_	64 (18.66)
	*T*_3_	229 (66.77)
	*T*_4_	41 (11.95)
N stage	*N*_0_	205 (59.77)
	*N*_1_	81 (23.61)
	*N*_2_	57 (16.62)
M stage	*M*_x_	47 (13.70)
	*M*_0_	246 (71.72)
	*M*_1_	50 (14.58)
Status	Alive	275 (80.17)
	Dead	68 (19.83)

### Different Gene Expression Profiles and Immunocyte Characteristics Between the Low- and High-TMB Groups

**FIGURE 4:**
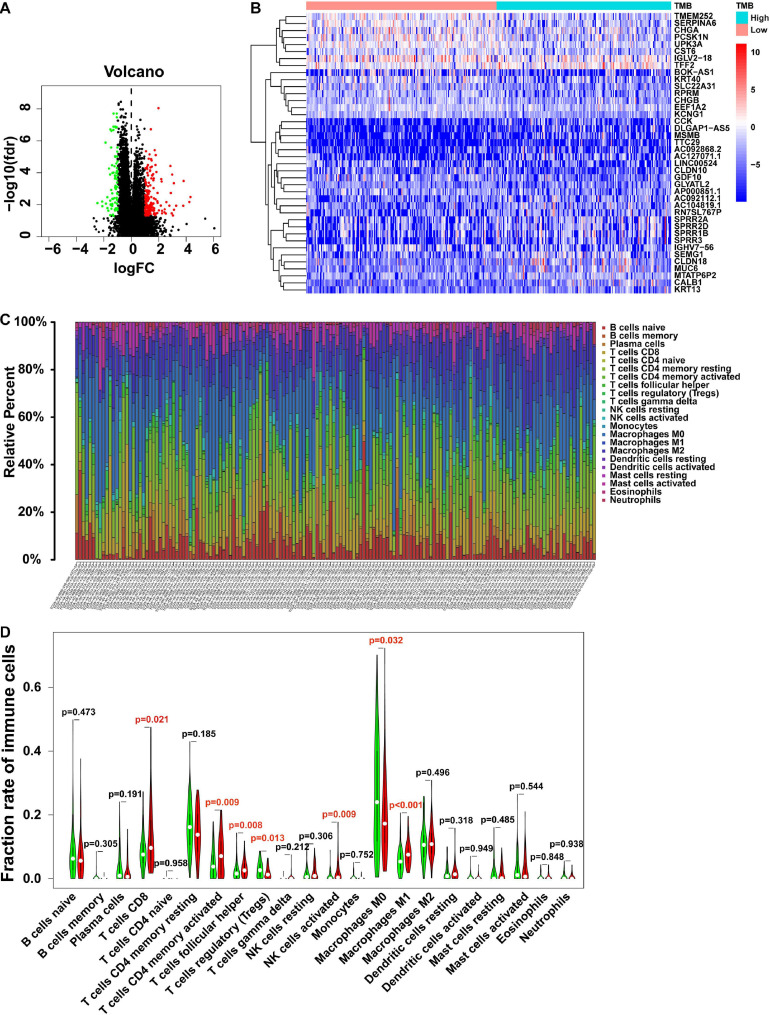
Gene expression profiles and immunocytes fraction between low- and high-TMB groups. **(A)** Volcano plot shows the DEGs in colon cancer. Red represents the high TMB group, and green represents the low TMB group. **(B)** Top 40 DEGs from low- and high-TMB groups are shown in the heatmap. **(C)** The fraction of 22 immunocyte subtypes of each colon cancer sample is represented by different colors listed on the right panel of the bar plot. **(D)** The violin plot shows the differences of infiltrated immunocyte subtypes between low- (green) and high-TMB (red) groups. The statistical significance is represented in red with *P*-value < 0.05. DEGs, differentially expressed genes.

To investigate the potential roles of TMB in the colon cancer process, we divided colon cancer patients into low- and high-TMB groups based on the medium value of TMB and analyzed DEGs in these two groups. With the cutoff value of | log_2_(FC)| > 1 and *P* < 0.05, the top 40 DEGs are shown in the heatmap (Figures 4A,B). Furthermore, KEGG pathway and GO enrichment analyses were also performed to explore potential signaling pathways and gene functions involved in tumor somatic mutations or immune responses (Tables 3, 4, respectively). Notably, the antigen processing and presentation pathway was enriched in the high-TMB group, suggesting a potential correlation between the TMB level and the immune response process.

**TABLE 3:** GESA for KEGG pathways between low- and high-TMB groups.

Group	Name	ES	NES	Nom *P*-value	FDR *q*-value
High TMB	KEGG ANTIGEN_PROCESSING_AND_PRESENTATION	0.684	1.948	0.004	0.125
Low TMB	KEGG_TIGHT_JUNCTION	–0.415	–1.654	0.014	0.221
	KEGG_PENTOSE_AND_GLUCURONATE_INTERCONVERSIONS	–0.570	–1.638	0.037	0.225
	KEGG_OTHER_GLYCAN_DEGRADATION	–0.655	–1.658	0.040	0.233
	KEGG_GLYCEROPHOSPHOLIPID_METABOLISM	–0.433	–1.664	0.006	0.245
	KEGG_BLADDER_CANCER	–0.440	–1.513	0.034	0.247

*ES, enrichment score; NES, normalized enrichment score; NOM *P*-value, normalized *P*-value; FDR *q*-value, false discovery rate *q*-value.*

**TABLE 4:** GESA for GO analysis between low- and high-TMB groups.

Group	Name	ES	NES	Nom *P*-value	FDR *q*-value
GO_CC TMB High	GO_POSTSYNAPTIC_CYTOSOL	0.587	1.629	0.033	0.245
GO_CC TMB Low	GO_LATERAL_PLASMA_MEMBRANE	–0.604	–2.093	0	0.069
	GO_APICOLATERAL_PLASMA_MEMBRANE	–0.728	–2.040	0	0.081
	GO_VACUOLAR_PROTON_TRANSPORTING_V_TYPE_ATPASE_COMPLEX	–0.701	–1.944	0.006	0.189
	GO_MULTIVESICULAR_BODY	–0.554	–1.817	0.004	0.231
	GO_DENDRITIC_SPINE_MEMBRANE	–0.702	–1.706	0.006	0.245
GO_BP TMB High	GO_MITOTIC_SISTER_CHROMATID_SEGREGATION	0.654	2.125	0.002	0.062
	GO_NEGATIVE_REGULATION_OF_METAPHASE_ANAPHASE_TRANSITION_OF_CELL_CYCLE	0.751	2.166	0	0.066
	GO_RESPONSE_TO_UV_C	0.751	2.135	0	0.070
	GO_NEGATIVE_REGULATION_OF_CHROMOSOME_SEGREGATION	0.727	2.192	0	0.084
	GO_REGULATION_OF_CHROMOSOME_SEGREGATION	0.636	2.045	0.006	0.100
GO_BP TMB Low	GO_REGULATION_OF_CYTOPLASMIC_TRANSPORT	–0.626	–1.911	0.008	0.240
	GO_ENERGY_RESERVE_METABOLIC_PROCESS	–0.494	–1.917	0	0.243
	GO_NEGATIVE_REGULATION_OF_EMBRYONIC_DEVELOPMENT	–0.730	–2.099	0	0.245
	GO_RENAL_SODIUM_EXCRETION	–0.685	–1.912	0.002	0.245
	GO_REGULATION_OF_WNT_SIGNALING_PATHWAY_PLANAR_CELL_POLARITY_PATHWAY	–0.731	–1.919	0.008	0.248
GO MF TMB High	GO_TRANSMEMBRANE_RECEPTOR_PROTEIN_SERINE_THREONINE_KINASE_BINDING	–0.817	–2.199	0	0.020
	GO_RECEPTOR_SERINE_THREONINE_KINASE_BINDING	–0.689	–2.062	0	0.064
	GO_ACTIVIN_BINDING	–0.765	–1.950	0	0.104
	GO_LYSOPHOSPHOLIPASE_ACTIVITY	–0.676	–1.958	0	0.111
	GO_VITAMIN_TRANSMEMBRANE_TRANSPORTER_ACTIVITY	–0.640	–1.971	0	0.119

*ES, enrichment score; NES, normalized enrichment score; NOM *P*-value, normalized *P*-value; FDR *q*-value, false discovery rate *q*-value.*

High TMB levels cause more neoantigens during the tumor process, leading to immune infiltration in the tumor microenvironment, which supports tumor initiation and development. In this study, the immunocyte characteristics were also investigated in different TMB groups. The relative percentages of 22 immunocyte subtypes of each colon cancer patient are exhibited by different colors in the box plot (Figure 4C). Furthermore, based on the Wilcoxon rank-sum test, CD8^+^ T cells, activated CD4^+^ memory T cells, follicular helper T cells, activated NK cells, and M1 macrophages showed higher fractions in the high-TMB group. In contrast, regulatory T cells (Tregs) and M0 macrophages accounted for a lower fraction in the high-TMB group (Figure 4D), which indicated that a high TMB promoted immunocyte infiltration in patients with colon cancer.

### The Immune Infiltration Profiles in Colon Cancer

**FIGURE 5:**
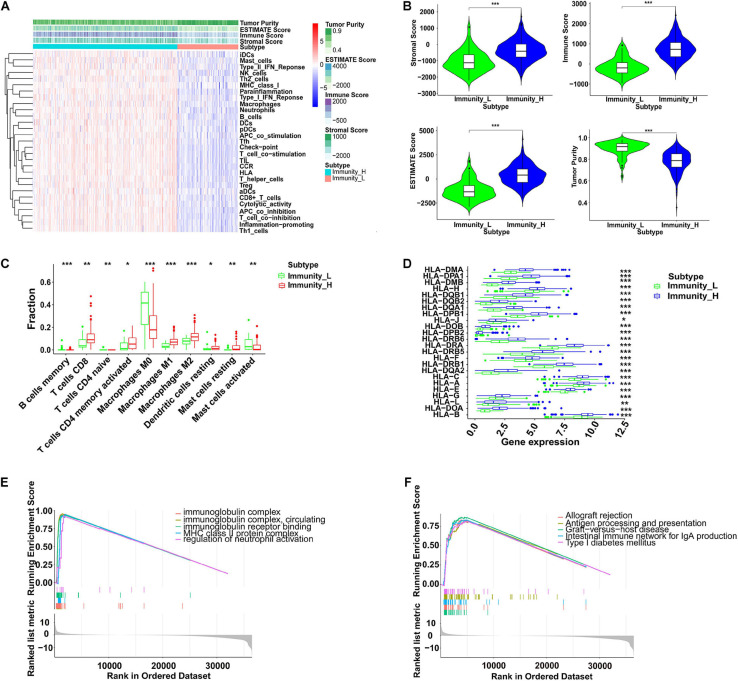
Immunity profiles analysis in colon cancer. **(A)** By ssGSEA, immune related gene sets were enriched in different immunity groups. The tumor purity, ESTIMATE score, immune score, and stromal score were calculated and are shown in the heatmap. **(B)** The violin plots indicates that the high immunity group showed a significantly higher stromal score, immune score, and ESTIMATE score, but lower tumor purity (***P < 0.001). **(C)** The fractions of 10 infiltrated immunocyte subtypes in different immunity groups (*P < 0.05, **P < 0.01, ***P < 0.001). **(D)** The RNA expression levels of HLA related genes in different immunity groups (*P < 0.05, **P < 0.01, ***P < 0.001). **(E)** GSEA of GO in different immunity groups. **(F)** GESA of KEGG in different immunity groups. ssGSEA, single sample gene set enrichment analysis; HLA, human leukocyte antigen; GO, gene ontology; KEGG, Kyoto Encyclopedia of Genes and Genomes.

To further explore immune infiltration profiles in patients with colon cancer, 29 immunocyte subtypes and immune-related pathways were analyzed by ssGSEA for each colon cancer sample (Figure 5A). With the division of low- and high-immunity groups from the TCGA data, using an unsupervised consensus clustering analysis, several parameters were applied to estimate the immune infiltration profiles, including tumor purity, ESTIMATE score, Immune score, and Stromal score. As shown in Figure 5B, the immune score, stromal score, and corresponding ESTIMATE score were higher in the high immunity group while the tumor purity was lower when compared with those in the low immunity group. The fractions of infiltrating immunocytes were significantly different between these two groups; memory B cells, naive CD4^+^ T cells, M0 macrophages, and activated mast cells were higher in the low-immunity group, whereas CD8^+^ T cells, activated memory CD4^+^ T cells, M1 and M2 macrophages, resting dendritic cells, and resting mast cells were increased in the high-immunity group, which suggests that more immune filtrations exist in the high-immunity group or in samples with low tumor purity (Figure 5C). In addition, the low immunity group exhibited significantly lower HLA related gene set expressions (Figure 5D).

To explore the biological behaviors among these immune subtypes, we performed GO and KEGG enrichment analyses. As shown in Figure 5E, the GO enrichment analysis revealed that a high immunity was related to the functions of the immunoglobulin complex, circulating immunoglobulin complex, immunoglobulin receptor binding, major histocompatibility complex class II (MHC-II) protein complex, and the regulation of neutrophil activation. The KEGG enrichment analysis showed that a high immunity was associated with allograft rejection, antigen processing and presentation, graft-versus-host disease, intestinal immune network for IgA production, and type I diabetes mellitus (Figure 5F). In addition, we also estimated the correlation between immune infiltration and MSI in colon cancer. As shown in Table 1, patients with high immune infiltration were more likely to be MSI-H (*P* < 0.001).

**FIGURE 6:**
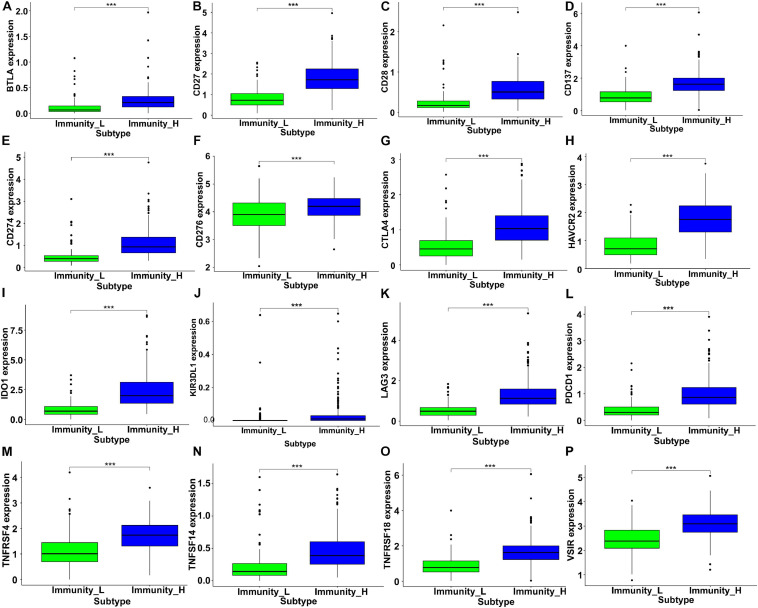
The expression levels of immune checkpoint related genes in different immunity groups of colon cancer. The gene expression of **(A)** BTLA, **(B)** CD27, **(C)** CD28, **(D)** CD137, **(E)** CD274, **(F)** CD276, **(G)** CTLA4, **(H)** HAVCR2, **(I)** IDO1, **(J)** KIR3DL1, **(K)** LAG3, **(L)** PDCD1, **(M)** TNFRSF4, **(N)** TNFSF14, **(O)** TNFRSF18, and **(P)** VSIR were significantly higher in the high immunity group (***P < 0.001).

As critical targets for immunotherapy, 16 immune checkpoint genes were assessed in the low- and high-immunity groups. The results indicated significantly increased expression levels among these checkpoint genes in the high-immunity group, including BTLA, CTLA4, IDO1, LAG3, and PDCD1 (Figure 6), which suggested that colon cancer patients in the high-immunity group could exhibit a better response to immune checkpoint inhibitors, such as CTLA4 and PD1 inhibiting reagents.

### Identification of Immune Related Genes and Their Prognostic Value in Patients With Colon Cancer

**FIGURE 7:**
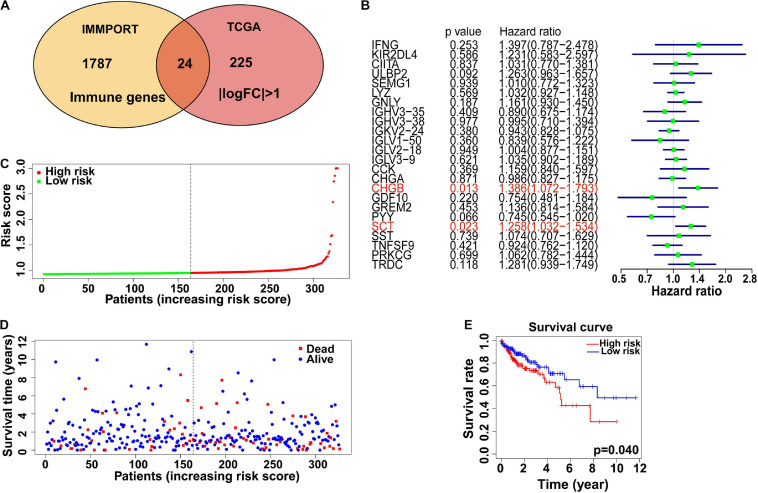
Immune-related genes and relations with colon cancer prognosis. **(A)** Venn plot shows core immune genes between the Immport and TCGA database. **(B)** Hazard ratio was analyzed among 24 identified immune-related genes by univariate Cox regression analysis. Two hub genes (CHGB and SCT) indicated significant difference in survival of colon cancer patients. **(C)** Immune risk score calculated for each colon cancer patients. Patients were divided into high risk and low risk group by median risk score. **(D)** The survival time of different colon cancer patients. **(E)** Low immune risk group showed better 5-year survival than High risk group.

Since immunocyte infiltration was promoted by high TMB levels in colon cancer based on previous data, we further explored the correlation between immune related genes and patient prognosis. Data from IMMPORT and TCGA were analyzed, and 24 immune related genes with | log_2_FC| > 1 were filtered as candidate risk genes to assess their correlation with prognosis (Figure 7A). In the univariate Cox analysis, two genes (CHGB and SCT) were shown to be correlated with the survival of patients with colon cancer (Table 5), while only SCT was found to be an independent prognostic factor based on the multivariate analysis (*P* < 0.001) (Figure 7B). In addition, we further investigated the expression level of SCT in 34 different tumors from the TIMER database and found that SCT expression was lower in colon cancer than in normal tissues (Supplementary Figure 2A). Furthermore, SCT expression showed significantly negative correlations with immunocyte infiltration, including B cell, CD8^+^ T cell, and neutrophil cell infiltration. Positive correlations also existed between SCT expression and CD4^+^ T cell and macrophages. However, there were no significant differences between tumor purity or dendritic cell infiltration with SCT expression (Supplementary Figure 2B). The colon cancer patients were then divided into low- and high-risk groups based on the median risk score (Figure 7C). Compared with patients in the low-risk group, those in the high-risk group indicated poorer survival probability based on our risk model (*P* = 0.04, Figures 7D,E), which suggested potential functions of the risk immune related genes in colon cancer prognosis.

**TABLE 5:** Univariate Cox regression analysis.

Gene	HR	Lower 95% CI	Upper 95% CI	Cox *P*-value
CHGB	1.386	1.072	1.793	0.013
SCT	1.258	1.032	1.534	0.023

*HR, hazard ratio; CI, confidence interval.*

### The Nomogram Model to Predict the Prognosis of Patients With Colon Cancer

**FIGURE 8:**
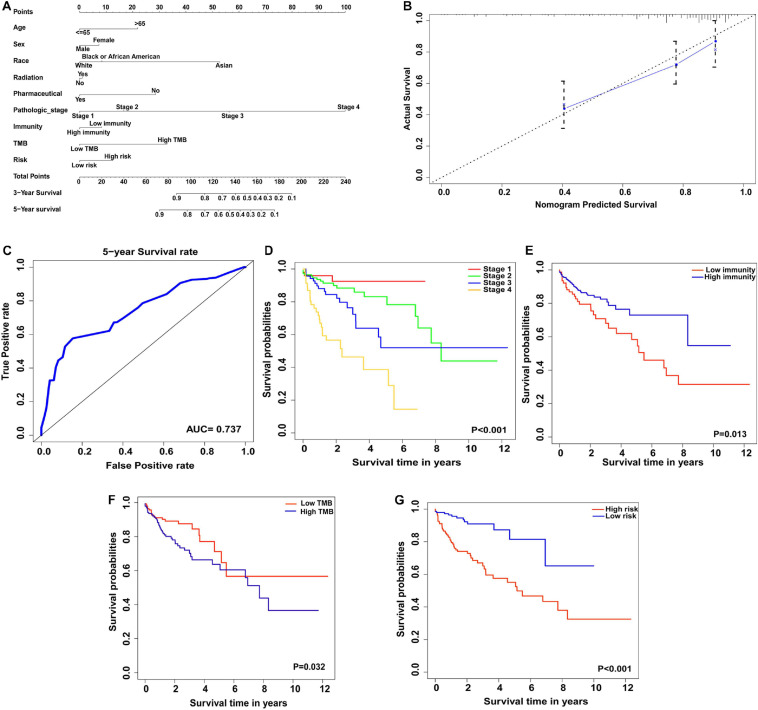
Construction of nomogram model for prognosis prediction of colon cancer patients. **(A)** Nomogram model to predict the prognosis of colon cancer based on nine clinicopathological factors. **(B)** Calibration plot of the nomogram model. The dotted line represents the ideal prediction ability. **(C)** ROC curve of 5-year survival of colon cancer based on nomogram model prediction. **(D–G)** The clinical stages, immunity level, TMB level and immunity risk were significantly correlated with 5-year survival in nomogram model.

For colon cancer patient management and therapeutic strategies, prognosis is a critical factor. In this study, combined with TMB profiles, immunocyte infiltration status and clinicopathological data, we constructed a nomogram model to predict the prognosis of colon cancer patients, which contained nine factors: age, sex, race, radiation therapy or pharmaceutical status, pathological stage, immunity status, immunity risk scores, and TMB. Each factor in the nomogram model was ascribed a weighted point that would be used to predict the survival of patients with colon cancer. In our nomogram model, being aged > 65 years was assigned 22 points, female was assigned 7 points, being Asian and African American were assigned 53 and 8 points respectively, having received radiation therapy was assigned 1 point, not having any pharmaceutical treatment was assigned 29 points, pathological stage 4 was assigned 100 points, being from a low-immunity group was assigned 8 points, being in a high-risk group was assigned 12 points, and having a high TMB level was assigned 33 points. The total points were used to predict the 3- or 5-year survival of colon cancer patients, and higher total points indicated a worse prognosis (Figure 8A). The performance of the nomogram model was then assessed by Harrell’s C-index, calibration plot and ROC curve analyses. Our nomogram model exhibited proper prediction accuracy and application potential for 5-year survival probability prediction with a close correspondence between the predicted curve and the actual survival plot, and a good C-index (0.764) and AUC (0.737) (Figures 8B,C). Notably, we further performed the Kaplan-Meier survival analyses and log-rank tests to estimate the survival differences between different pathological stages (Stage 1–4), immunity status (low or high immunity), TMB levels (low or high TMB), and immune gene signature based-risk groups (low or high risk). As shown in Figure 8D, the survival prognosis of colon cancer patients with higher pathological stages were worse than those with lower ones (*P* < 0.001). Patients with high immunity or low TMB were found to have better prognoses (*P* = 0.013 and 0.032, respectively) (Figures 8E,F). In the immune gene signature based-risk model, patients with high-risk scores had poorer prognoses (*P* < 0.001) (Figure 8G).

## Discussion

As the most frequent malignancy in the digestive system, colon cancer exhibits a high incidence and mortality, but unfortunately, has a poor prognosis and unsatisfactory therapeutic outcomes. With the improvements in imaging techniques to detect metastatic lesions, adjuvant multidisciplinary therapies for resectable stage III patients and neoadjuvant therapeutic strategies for local advanced colon cancer patients, the 5-year survival rates of all stages have increased from 51% (mid-1970s) to 66% (2006–2012) (Siegel et al., 2017; Howlader et al., 2020). In addition, for patients with distant metastasis, the 2-year survival rate increased from 21% (1989–1992) to 35% (2009–2012) (Surveillance, Epidemiology, and End Results [SEER], 2016). Thus, the exploration and development of proper therapies for advanced metastatic colon cancer patients are quite urgent and necessary. Since the discovery and successful application of immune checkpoint inhibitors or ICBs, such as ipilimumab and pembrolizumab, in metastatic melanoma and NSCLC, immunotherapy has become a potential choice for advanced cancer patients with distant metastasis (Garon et al., 2015; Robert et al., 2015). In 2017, immune checkpoint therapy was approved by the U.S. Food and Drug Administration for patients with dMMR-MSI-H CRCs (Ganesh et al., 2019). However, dMMR-MSI-H only accounts for approximately 15% of CRC patients. The detailed immune response mechanisms and effectiveness of ICBs are still controversial. In this study, we analyzed somatic mutation, MSI, immunocyte infiltration, transcriptome, and clinicopathological data from the public TCGA database and investigated the correlations between TMB, immune-related genes, immunocyte infiltration, and colon cancer progression.

Somatic mutations always occur during cancer progression and are accompanied by mutated gene transcription, translation, and neoantigen peptide synthesis. Part of the neoantigens will then be processed and presented on the cell surface with MHC, which will be recognized and targeted by the immunocytes (Rizvi et al., 2015; Riaz et al., 2016; Chan et al., 2019). To quantify the somatic mutations, TMB was defined as the number of synonymous and non-synonymous mutations per million bases, including silent mutations, missense mutations, insertions or deletions, and copy number gains and losses. In tumors, TMB was found to be positively correlated with tumor neoantigen burden (Chan et al., 2019). Presenting variability among different types of tumors, TMB was found to be high in melanoma, NSCLCs, and squamous carcinomas, while in leukemias and some pediatric tumors, TMB was low (Alexandrov et al., 2013; Chalmers et al., 2017). In our study, we analyzed the mutation landscape in colon cancer samples from the TCGA database and found that missense mutations were the most frequent variant class, and that C > T was the major variant type for single nucleotides (Figures 1A,C). Additionally, we found the top mutated genes and their interactions, including APC, TP53, TTN, and KRAS, suggesting their critical roles in colon cancer carcinogenesis (Figures 1F, 2) (Kwong and Dove, 2009; Wolff et al., 2018; Nakayama and Oshima, 2019). Furthermore, the TMB level was correlated with clinicopathological parameters in the matched colon cancer patient cohort, including AJCC stage, N stage, and M stage, indicating that the TMB level could be a risk factor in colon cancer development (Table 2 and Figure 3).

Several detection methods were applied in clinical trials to evaluate the level of TMB from samples of patients with a malignancy. Including whole genome sequencing and whole exome sequencing (WES), the next-generation sequencing technique is used to detect genomic alterations (Büttner et al., 2019). As the WES covered the coding region of genes in the genome, it was used in many clinical trials to evaluate the TMB status in different types of cancer and is currently considered as a reference standard (Hugo et al., 2016; Carbone et al., 2017; Büttner et al., 2019). In CRC, the WES was applied to assess the TMB to investigate the response to pembrolizumab, one of the ICBs used in immunotherapy (Le et al., 2015). Furthermore, targeted gene panels focusing on cancer-related genes were also developed as an alternative technique to the WES in recent years, such as FoundationOne, FoundationOne CDx, and MSK-IMPACT (Powles et al., 2018; Büttner et al., 2019; Chang et al., 2019). However, the gene panel data of CRC is limited, more clinical trials are needed in future investigations.

Microsatellites, which are prone to being DNA replication error sites, refer to short, tandemly repeated sequences of mononucleotide, dinucleotide, or nucleotide repeats located in the genome (Yamamoto and Imai, 2015; Baretti and Le, 2018). As additional bases insertion or existing bases deletion from microsatellites, DNA mismatch errors occur during DNA replication, which can be supervised and corrected by the MMR system (Arana and Kunkel, 2010). For patients with MMR deficiency (dMMR), accumulated mismatch mutation and frameshift mutation will lead to the MSI phenotype, neoantigens production, and is related to carcinogenesis of several cancers, such as CRC, gastric cancer, pancreatic cancer, and endometrial cancer (Palomaki et al., 2009; Seo et al., 2009; Ghidini et al., 2020; Lin et al., 2020). In CRC, dMMR or high levels of MSI (MSI-H) were correlated with high TMB and immunocyte infiltration, which made dMMR-MSI-H CRC responsible for ICBs (Alexander et al., 2001; Llosa et al., 2015; Ganesh et al., 2019). Consistent with previous studies, our study indicated the positive correlation between TMB and MSI levels based on the analyses of transcriptome data from patients with colon cancer (Table 1).

Due to the high frequency of neoantigens in the high-TMB group, immune responses such as immunocyte infiltration would be more active. We investigated the percentage of 22 different subtypes of infiltrated immunocytes in each colon cancer patient and found that CD8^+^ T cells, activated CD4^+^ memory T cells, activated NK cells, and M1 macrophages were increased in the high-TMB group, indicating that the immunocyte-killing activity was increased in high-TMB colon cancer patients (Figures 4C,D), which is consistent with recent studies in gastrointestinal system malignancies (Zappasodi et al., 2018; Eso and Seno, 2020). Clinically, the cancer-related immune status is difficult to estimate due to limited information about cancer specific antigens. Recent studies suggest that similar T cell receptor (TCR) sequences could be clustered to identify cancer antigen-specific TCRs and evaluate immune status based on cancer genomics sequencing, which indicates new strategies for precise immunotherapy assessment (Zhang et al., 2020).

To further reveal the immune infiltration profiles, we investigated immunocytes and immune related genes and pathways by ssGSEA from transcriptome data and found different immune infiltration profiles among the low- and high-immunity groups divided by CIBERSORT (Figures 5A–D). In addition, immunoglobulin complex related functions and MHC-II complexes were enriched in the GO analysis. Allograft rejection, antigen processing, and presentation were the top ranked KEGG enriched pathways, indicating their contributions to colon cancer immunity (Figures 5E,F). Notably, the expression of immune checkpoint genes, such as CTLA4 and PDCD1 (PD-1), was increased in the high-immunity groups, suggesting the potential therapeutic targets for ICBs application (Figure 6).

Immune-related genes were analyzed by combining the TCGA and IMMPORT databases. Two hub genes were revealed to be critical in the immunocyte infiltration mechanism in colon cancer by a univariable Cox regression model (Figures 7A,B). CHGB (chromogranin-B, CgB), which colocalizes with CHGA, is expressed in secretory granules of neuroendocrine cells and the function of CHGB is still limited. Paul et al. suggested that CHGB could be a prognostic marker in neuroendocrine tumors (Wanigasekara et al., 2015). Secreted by endocrine S cells in the proximal small intestinal mucosa, SCT (secretin) encoding preproprotein is involved in the regulation of duodenal pH, food intake, and water homeostasis (Afroze et al., 2013). Onori et al. (2010) found that SCT could inhibit the growth of cholangiocarcinoma *via* the cAMP-dependent signaling pathway, indicating its regulatory roles in gastrointestinal cancers. In this study, we further analyzed hub genes by a multivariable Cox regression model. We found that the expression level of SCT was significantly lower in colon cancer samples than in normal samples, and its correlation with immunocyte infiltration, suggested that SCT could be a critical gene in colon cancer development and tumor related immunocyte infiltration (Supplementary Figure 2). In general, these hub genes play roles in immunoglobin variation, immunocyte receptor constant, antigen recognition, and macrophage differentiation. However, the mechanistic details of these hub genes require further exploration.

Recent studies revealed that the responses and outcomes of ICBs were related to immunocyte infiltration in several types of cancers, including gastric cancer, breast cancer, CRC, and esophageal cancer (Simpson et al., 2010; Hu et al., 2017; Wei et al., 2018; Huang and Fu, 2019). In CRC, dMMR-MSI-H was considered to be a biomarker for the response to ICBs. However, limited by the low percentage of dMMR-MSI-H patients (∼15%), more precise prediction biomarkers are needed. In our study, we developed a novel nomogram prediction model based on data from the TCGA database. Nine clinicopathological factors were enrolled in our nomogram model: age, sex, race, radiotherapy and pharmacotherapy status, pathological stage, immunity status, TMB, and immunity risk (Figure 8A). Estimated by each factor in the nomogram model, the survival of patients with colon cancer was predicted by the nomogram total points, in which higher points meant a worse prognosis. Consistent with previous studies of tumor immunity, high immunity status in our nomogram model showed survival improvement in patients with colon cancer (Figure 8E) (Galon et al., 2006; Di Caro et al., 2014; Topalian et al., 2016). Notably, in our nomogram model, colon cancer patients with low TMB exhibited higher overall survival than high TMB patients, whereas high TMB predicted improved survival in patients with melanoma (Figure 8F) (Lauss et al., 2017). Due to the heterogeneous profiles of colon cancer, the correlation between TMB and patient survival, and the mechanisms of immunocyte infiltration in different immunotherapy protocols require further exploration.

## Conclusion

In this study, we investigated the patterns of TMB and immunocyte infiltration in patients with colon cancer based on the TCGA database, which may provide valuable clues of immunotherapy for colon cancer. In addition, we established a nomogram model integrating TMB and immune infiltration with remarkable performance in prognosis prediction, indicating its potential application in the management of colon cancer patients.

## Data Availability Statement

The datasets presented in this study can be found in online repositories. The names of the repository/repositories and accession number(s) can be found in the article/Supplementary Material.

## Author Contributions

All authors participated in the design of this study, and read and approved the final version of the manuscript. ZZ, XX, and XW were in charge of data obtainment and analysis. XZ, WL, and TS obtained the data. YC and JW were in charge of statistical analysis. ZZ and XX drafted the manuscript. CD and HZ supervised this study and revised the manuscript.

## Conflict of Interest

The authors declare that the research was conducted in the absence of any commercial or financial relationships that could be construed as a potential conflict of interest.

## Footnotes

1 https://portal.gdc.cancer.gov/

2 https://github.com/PoisonAlien/maftools

3 http://software.broadinstitute.org/gsea/msigdb/

4 http://cibersort.stanford.edu/

5 https://cistrome.shinyapps.io/timer

## Supplementary Material

Supplementary Figure 1 The workflow of this study.

Supplementary Figure 2 The expression of SCT and its correlation with immunocytes infiltration. **(A)** The expression level of SCT in 32 different type of tumors and matched normal samples. ^∗^*P* < 0.05, ^∗∗^*P* < 0.01, ^∗∗∗^*P* < 0.001. **(B)** The correlation of SCT expression level and immunocytes infiltration status of colon cancer based on the TIMER database validation.
