# Supplementary figures and images for: Correlations Between Tumor Mutation Burden and Immunocyte Infiltration and Their Prognostic Value in Colon Cancer
Source: Front Genet. 2021 Feb 16;12:623424. doi: 10.3389/fgene.2021.623424 (PMC7921807; doi:10.3389/fgene.2021.623424)

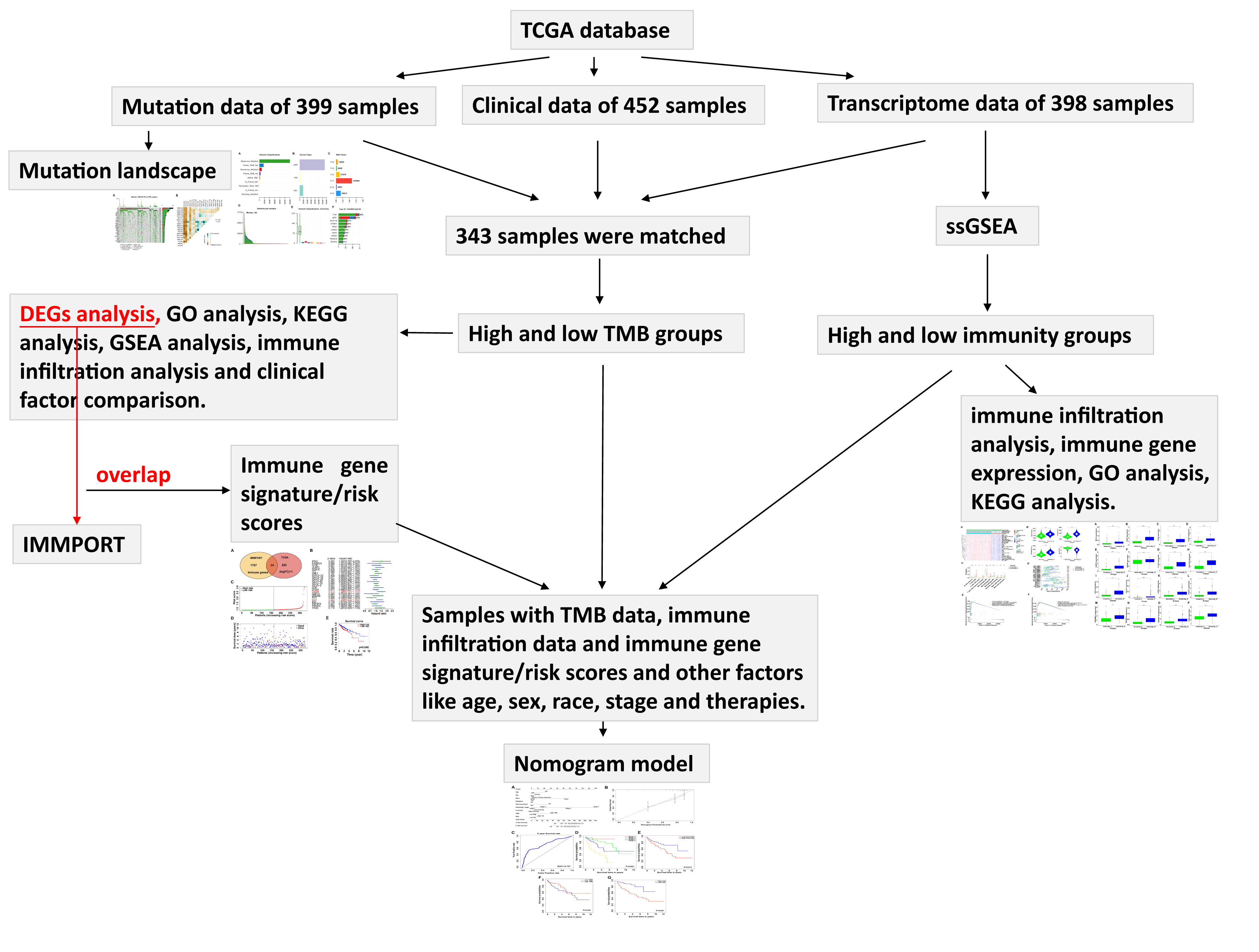

Supplement: Supplementary Figure 1 — The workflow of this study. [file Image_1.TIF]

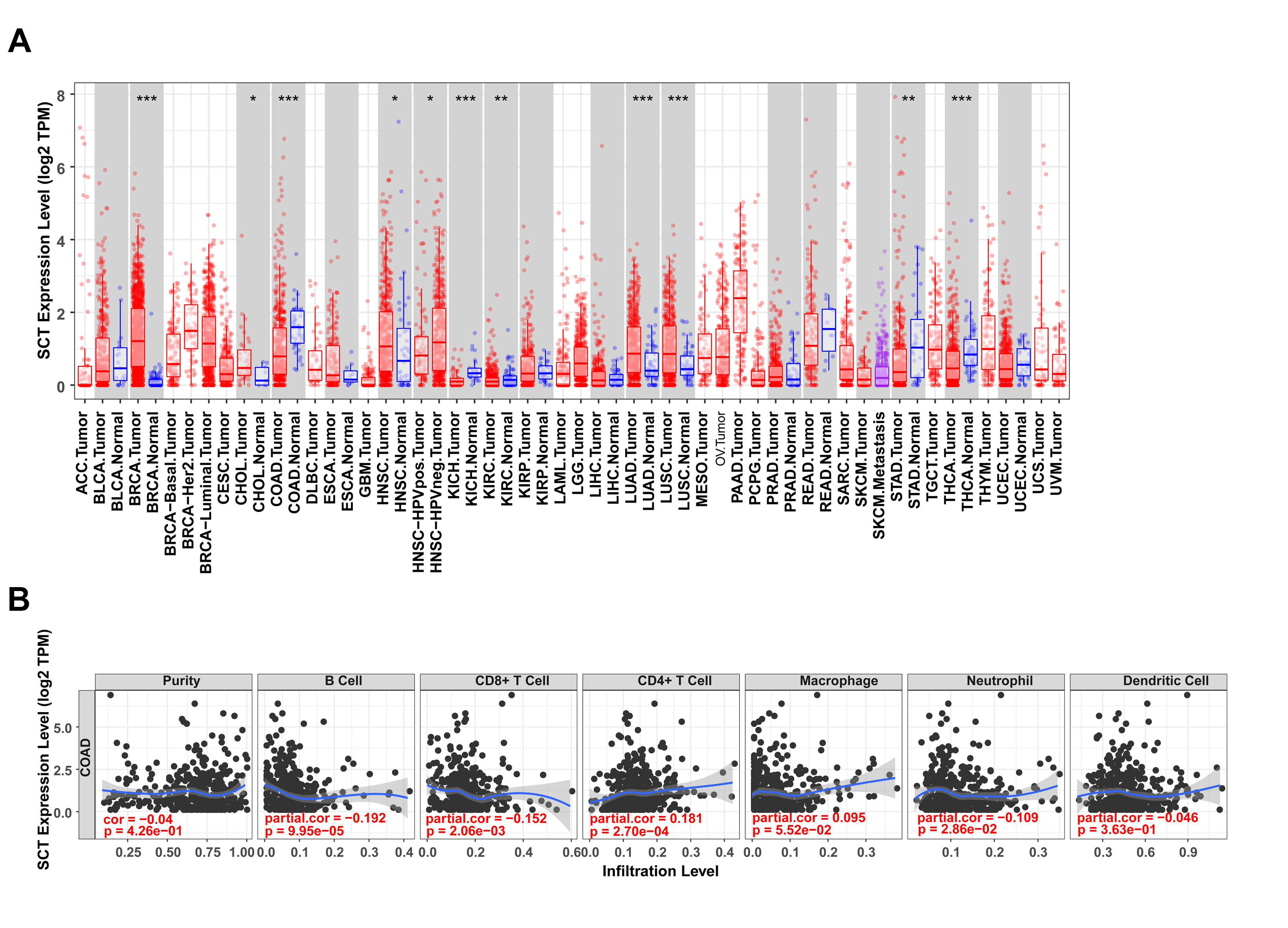

Supplement: Supplementary Figure 2 — The expression of SCT and its correlation with immunocytes infiltration. (A) The expression level of SCT in 32 different type of tumors and matched normal samples. ∗P < 0.05, ∗∗P < 0.01, ∗∗∗P < 0.001. (B) The correlation of SCT expression level and immunocytes infiltration status of colon cancer based on the TIMER database validation. [file Image_2.TIF]
